# Bacterial Resistances and Sensibilities in a Tertiary Care Hospital in Romania—A Retrospective Analysis

**DOI:** 10.3390/microorganisms12081517

**Published:** 2024-07-24

**Authors:** Lazar Chisavu, Flavia Chisavu, Luciana Marc, Adelina Mihaescu, Flaviu Bob, Monica Licker, Viviana Ivan, Adalbert Schiller

**Affiliations:** 1 Centre for Molecular Research in Nephrology and Vascular Disease, Faculty of Medicine “Victor Babes”, Eftimie Murgu Square No. 2, 300041 Timisoara, Romania; chisavul.lazar@umft.ro (L.C.); farkas.flavia@umft.ro (F.C.); mihaescu.adelina@umft.ro (A.M.); bob.flaviuraul@umft.ro (F.B.); ivan.viviana@umft.ro (V.I.); schiller.adalbert@umft.ro (A.S.); 2 Discipline of Nephrology, University of Medicine and Pharmacy “Victor Babes”, Eftimie Murgu Square No. 2, 300041 Timisoara, Romania; 3 “Louis Turcanu” Emergency County Hospital for Children, 300011 Timisoara, Romania; 4 Microbiology Department, Multidisciplinary Research Center of Antimicrobial Resistance, “Victor Babes” University of Medicine and Pharmacy, 300041 Timisoara, Romania; licker.monica@umft.ro; 5 Microbiology Laboratory, “Pius Brinzeu” County Clinical Emergency Hospital, 300723 Timisoara, Romania; 6 Discipline of Cardiology, University of Medicine and Pharmacy “Victor Babes”, Eftimie Murgu Square No. 2, 300041 Timisoara, Romania

**Keywords:** antibiotic resistance, uroculture, blood culture, bronchial aspirate culture, *Escherichia coli*, *Staphylococcus aureus*, *Klebsiella*

## Abstract

The increase in bacterial resistance is currently a global burden for the health care system. In order to evaluate the resistance rates of several bacteria from the most encountered cultures in clinical practice, we performed a retrospective analysis of all of the positive cultures from the year 2021 in a tertiary care hospital in Romania. Our analysis captured 3299 positive cultures. The median age of the patients was 62 years (IQR: 41–71 years old) with a slight predominance among females (53.1%). Overall, the most common cultures were urocultures, wound secretion cultures and blood cultures, and the most common identified bacteria were *Escherichia coli*, *Staphylococcus aureus* and *Klebsiella* spp. Positive cultures with the highest resistance rates were found in the bronchial aspirate cultures, catheter tip cultures, urocultures and blood cultures. *Escherichia coli* (n = 996) had the highest resistance to ampicillin (19.8%) and trimetoprim-sulfametoxazole (16.4%), while *Staphylococcus aureus* (n = 698) presented the highest resistance rates to clindamycin (27.4%) and oxaciline (19.7%). *Klebsiella* (n = 481) presented the highest resistance rates to piperaciline-tazobactam (25.2%) and ampicillin (20.4%), whereas *Acinetobacter baumanii* (n = 123) presented a resistance rate of more than 50% to carbapenems, gentamicin, ciprofloxacin and ceftazidime. The aim of our study was to identify bacterial resistance rates in order to provide updated clinical data to guide physicians in choosing the best empirical antibiotic treatment, especially in the west part of Romania.

## 1. Introduction

Antimicrobial resistance is one of the hottest topics of this millennium. Current studies show an ascending trend of overall bacterial resistance, which extends even to “reserve antibiotics”, with impacts on quality of life, hospitalization and mortality [1,2,3,4,5,6,7]. The Review on Antimicrobial Resistance from the United Kingdom (UK) Government, World Health Organization (WHO) and many other organizations agree that there is an imperative need for a mutual approach in order to reduce bacterial resistance [1,8,9,10,11,12]. The latest predictions suggest that by 2050, resistant bacteria could kill more than 10 million people per year [1].

Antimicrobial resistance is heterogeneous regarding geographic regions. The first world-wide estimates on the burden of antimicrobial resistance were published in 2022 in Lancet [1]. This study estimated that almost 5 million people died in 2019 due to bacterial infections, and 25% of these patients presented a death attributable to bacterial antimicrobial resistance [1]. The pathogens identified as the leading causes of death were *Escherichia coli*, *Staphylococcus aureus*, *Klebsiella pneumonia*, *Streptococcus pneumonia*, *Acinetobacter baumanii* and *Pseudomonas aeruginosa* [1]. In a published study on eight bacterial pathogens and 16 pathogen–drug combinations from the European Union, Cassini et al. identified the same germs as the leading causes of antimicrobial resistance-associated mortality [2].

Data from Romania are scarce regarding bacterial resistance, and the majority of studies focus on one germ or just one site of infection [13,14,15,16,17,18]. On the other hand, hospital-associated infection, which is the leading cause of multidrug-resistant infections, seems to be underreported in Romania [16]. 

In order to address this issue and create a better image of bacterial resistances and sensibilities in Romania, we performed a retrospective analysis of all of the positive cultures from a tertiary care hospital in the west part of Romania. The data were extracted over twelve months, representing the year 2021. We present the sensibilities and resistances stratified by positive cultures and by the identified bacteria.

## 2. Materials and Methods

We performed a cross-sectional retrospective analysis of all positive cultures from the Emergency County Hospital “Pius Brinzeu” from Timisoara, Romania from the year 2021. This study was performed in accordance with the Ethics Code of the World Medical Association and the “Pius Brinzeu” Emergency County Hospital, and the Timisoara Ethics Committee approved this study (466/17 May 2024). This study followed the Declaration of Helsinki recommendations. The patients signed an informed consent form upon admission to the hospital. We used the electronic data system in order to extract all of the positive cultures. We recorded the age, gender, the culture type and the identified bacterial pathogen. In addition, we extracted the sensibilities and resistance rates to several tested antibiotics.

### 2.1. Sample Collection

The samples were collected over a 12-month period, comprising all of the positive cultures from the admitted patients used to confirm the clinical suspicion of infection. Cultures were performed according to the working protocol of the Microbiology Laboratory of Emergency County Hospital “Pius Brinzeu” in Timisoara.

### 2.2. Bacterial Identification and Antibiotic Testing

Cultures were performed according to the working protocol of the Microbiology Laboratory of Emergency County Hospital “Pius Brinzeu” from Timisoara. All isolates were first identified using the VITEK® 2 GN and VITEK® 2 GP ID cards (BioMérieux, Marcy l’Etoile, France). For *Staphylococcus aureus* and *Streptococcus* spp. identification, latex agglutination tests were also conducted using the Staphytect Plus and Streptococcal Grouping kit (Thermo Fischer Scientic). Antimicrobial susceptibility tests (AST) were performed using the VITEK 2 GN AST-N222 and VITEK 2 AST GP 67 cards (BioMérieux, Marcy l’Etoile, France) by the determination of the minimum inhibitory concentration (MIC) and the Kirby–Bauer disk diffusion method [19,20]. Classification into resistance phenotypes was performed according to the Clinical Laboratory and Standards Institute (CLSI) criteria [20]. We used the following reference strains: *Escherichia coli* ATCC 25922, *Klebsiella pneumoniae* ATCC 1705, *Pseudomonas aeruginosa* ATCC 27853 and *Staphylococcus aureus* ATCC 25923. For the Kirby–Bauer disk diffusion method, antibiotics from Thermo Fischer Scientific were used. The concentrations of the antibiotic disks were chosen according to the CLSI 2021 standard [20].

### 2.3. Culture Types and Identified Bacteria

The identified cultures were blood cultures, wound secretion cultures, peritoneal liquid cultures, cervical cultures, vaginal cultures, urocultures, sputum cultures, bronchial aspirate cultures, catheter tip cultures and abcess cultures.

The germs that we identified and evaluated were *Streptococcus*, *Pseudomonas aeruginosa*, *Enterobacter* spp., *Staphylococcus aureus*, *Bacillus cereus*, *Acinetobacter baumanii*, *Serratia marcesnens*, *Providencia*, *Proteus mirabilis*, *Corynebacterium*, *Citrobacter*, *Escherichia coli* and *Klebsiella* spp. 

The outcomes of our study are the bacteria distribution regarding the culture type and the incidence of certain antibiotics’ resistance and sensibilities. 

The recorded antibiotics are presented in Table 1.

### 2.4. Statistical Analysis

Data are presented as numbers and percentages for categorical variables. The age is presented as median (M) and interquartile range (IQR) because the age distribution was non-Gaussian. The evaluation of distribution was performed using Shapiro–Wilk test. The test used was the Chi-square test for categorical variables. A *p*-value less than 0.05 was considered statistically significant. The analysis was performed using MedCalc® Statistical Software version 22.021 (MedCalc Software Ltd., Ostend, Belgium; https://www.medcalc.org; Accessed on 19 May 2024). In the first part of the analysis, we evaluated the incidences of sensibilities and resistance stratified by the culture type, and in the second one, we stratified the data by bacterial type. 

One should mention that the antibiograms were not uniform regarding bacteria, and not even for the same bacteria. For instance, *Escherichia coli* may not be tested for the same antibiotic panel in different patients; *Klebsiella* and *Staphylococcus aureus* were tested for different antibiotics. One should keep in mind these aspects when interpreting the results. All of the percentages refer to the entire number of bacteria.

## 3. Results

### 3.1. Age Distribution

During the year 2021, the total number of admissions in our hospital was 41,027. The number of positive cultures was 3658. The median age of patients with positive cultures was 62 years (IQR: 41–71 years) with a slight predominance in the female gender (53.1%). A number of 317 positive cultures were identified in children, with a median age of 2 years (IQR = 0–8 years). We stratified the ages of the patients by decades, and the extended results are presented in Table 2.

### 3.2. Culture and Bacteria Distribution

We identified the number of cultures stratified by the type of the cultures as presented in the Materials and Methods Section. Overall, out of the 3658 positive cultures identified over the twelve-month period of this study, we excluded 320 cultures from the current analysis due to the heterogeneous distribution across ages (pharyngeal exudates, nasal exudates, tegument cultures, amniotic liquid cultures, etc.). In this analysis, the total number of studied cultures was 3338. The most common positive cultures were the urocultures, wound secretion cultures and blood cultures, accounting for 73.12%.

We evaluated the distributions of the identified germs, with the most common ones being *Escherichia coli*, *Staphylococcus aureus* and *Klebsiella* spp. (65.14%). We retrieved data regarding 3299 bacteria. We excluded positive fungal cultures from our evaluation.

Bacteria distribution regarding culture type was different. For instance, in blood cultures, the most common identified germ was *Staphylococcus aureus*—as also seen in the wound secretion cultures—while in the urocultures, the most common one was *Escherichia coli*. The extended results are presented in Table 3.

### 3.3. Resistance Rates and Sensibilities Stratified by Cultures

The sensibilities and resistance rates stratified by cultures are presented in Supplementary Tables S1 and S2.

**Figure 1:**
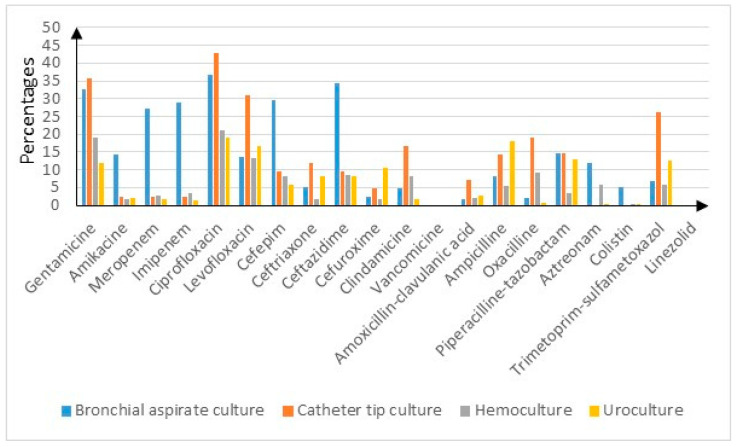
Antibiotic resistance rates for bronchial aspirate cultures, catheter tip cultures, blood cultures and urocultures.

The overall resistance rates were low, ranging from 0% (vancomycin and linezolid) to 16.9% (ciprofloxacin). The highest resistance rates were found in bronchial aspirate cultures, catheter tip cultures, urocultures and blood cultures—as shown in Figure 1. For instance, in the bronchial aspirate cultures, the resistance rates for gentamycin and ceftazidime (*p* < 0.05) were higher than 30%, while penem resistance rates were encountered in over 25% of the cultures (*p* < 0.05). The catheter tip cultures presented the highest resistance rates for gentamycin—35.7%—followed by ciprofloxacin (42.9%) and levofloxacin (31%) (*p* < 0.05). The urocultures had the highest resistance rates for ciprofloxacin—19%—and ampicillin—18% (*p* < 0.05). The blood cultures were most likely resistant to gentamycin—19.2%—and ciprofloxacin—21.1% (*p* < 0.05).

As expected, the cultures with the highest overall resistance rates presented the lowest sensibilities and vice versa—as seen in Supplemental Table S2.

### 3.4. Resistance Rates and Sensibilities Stratified by Bacteria

**Figure 2:**
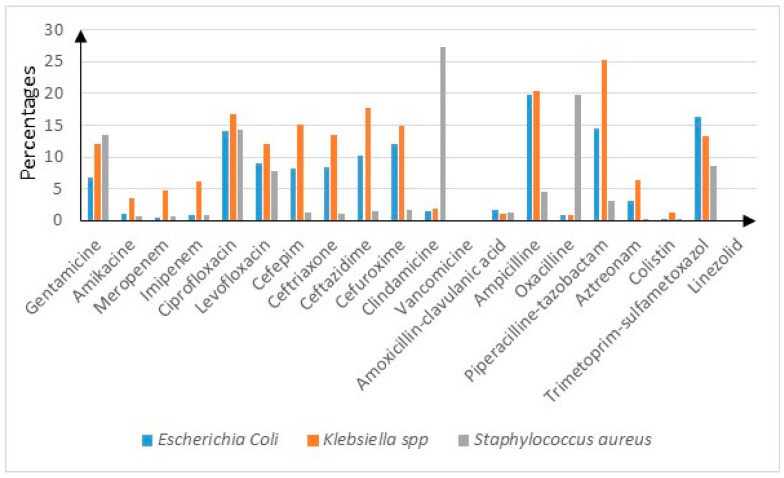
*Escherichia coli*, *Klebsiella* spp. and *Staphylococcus aureus* antibiotic resistance rates.

To complete the analysis, we evaluated the sensibilities and resistance rates using bacterial pathogen classification—as shown in Supplemental Tables S3 and S4. *Escherichia coli* presented the highest resistance to ampicillin (19.8%), trimethoprim-sulfamethoxazole (16.4%) and piperacillin-tazobactam (14.5%) (*p* < 0.05) *Staphylococcus aureus* presented the highest resistance rates to clindamycin (27.4%) and oxacillin (19.7%) (*p* < 0.05). One should mention that no *Staphylococcus aureus* cultures were resistant to vancomycin nor linezolid. *Klebsiella* spp. presented the highest resistance rates to piperacillin-tazobactam (25.2%), ampicillin (20.4%) and cefuroxime (14.8%) (*p* < 0.05) Figure 2. High resistance rates were identified in *Providencia* (around 30% for Penems and 50% for aminoglycosides, and almost 50% for ceftazidime and piperacillin-tazobactam) (*p* < 0.05), *Acinetobacter baumanii* (>50% for penems, gentamycin, ciprofloxacin and ceftazidime) and *Corynebacterium* (around 50% for levofloxacin, ceftriaxone, clindamycin and ampicillin) (*p* < 0.05). The highest resistance rates for *Pseudomonas aeruginosa* were to piperacillin-tazobactam (16.9%), ceftazidime (14.6%) and imipenem (12.9%) (*p* < 0.05). *Streptococcus* spp. presented higher resistance rates only to clindamycin (24.4%) and oxacillin (7.9%) (*p* < 0.05). *Enterobacter* spp. presented higher resistance rates to ceftazidime (20%) and cefepime (15%) (*p* < 0.05). *Proteus* presented higher resistance rates to ampicillin (18.3%) and trimethoprim-sulfamethoxazole (16.9%) (*p* < 0.05).

## 4. Discussions

In this study, we evaluated the distribution of bacterial pathogens in different cultures. The aim of our analysis was to describe the trend of antimicrobial patterns in patients with bacterial infections. Thus, we stratified the positive cultures firstly by the bacterial pathogen, and secondly by the bacterial sensibility and resistance pattern in different positive cultures. This study represents the mirroring of one-year bacterial infections in the west part of Romania that focused on the resistance trends of all bacterial pathogens encountered in all age groups.

The median age of the patients was 62 years, with less than 10% being minors. For instance, Axente reported a mean age of 60 years in Romania [21], and Mayr reported 63.1 years in the USA [22].

The most common encountered positive cultures were urocultures, wound secretion cultures and blood cultures. The study by Arbune on ESKAPE pathogens (*Enterococcus faecium*, *Staphylococcus aureus*, *Klebsiella pneumoniae*, *Acinetobacter baumannii*, *Pseudomonas aeruginosa* and *Escherichia coli*) from an infectious disease hospital in Romania reported a similar distribution in positive cultures [13].

Bacterial distribution following positive cultures seems to follow an international pattern, with some differences regarding certain regions. For instance, the most common identified bacteria in blood cultures was *Staphylococcus aureus* in Australia [23] as well as in the USA [24], while in China, *Escherichia coli* was the leading pathogen [25]. A recent review on urinary tract infections showed that *Escherichia coli* remains the most encountered bacteria in urocultures, as seen in our study [26]. Regarding wound secretion cultures, *Staphylococcus aureus* is the most common bacteria worldwide [27,28,29].

The culture resistance and sensibility distributions represent the highlights of our study. We identified the highest resistance rates in bronchial aspirate cultures. One should expect these results, as the bronchial aspirate culture is most likely performed in intensive care unit settings (ICU). The majority of these patients were intubated, as they were undergoing an invasive procedure, putting them at a higher risk of multidrug-resistant infection. Intensive care units are prone to multidrug-resistant bacteria due to the high use of antibiotics and even reserve antibiotics, especially in septic patients. In a recent study by Han, and even in an older one by Wroblewska on patients in the ICU setting, most of the identified microbes were multidrug resistant, with most of the bacteria being *Acinetobacter*, *Staphylococcus*, *Pseudomonas* and *Klebsiella*, just like in our study [30,31].

The high resistance rate to ciprofloxacin in positive urocultures is mainly secondary to the wide use of quinolones as a first-line agent in treating urinary tract infections in the study region. A relatively recent analysis regarding positive uroculture resistance in six countries in Eastern and Northern Europe show that countries analyzed in Eastern Europe present the highest resistance rates to ciprofloxacin (Russia and Poland) [32]. On the other hand, one should keep in mind that all of our positive cultures were in hospitalized patients and not outpatients, increasing the probability that some of the patients developed positive urocultures secondary to urine catheter insertion.

This kind of stratification regarding resistance is possibly helpful for clinicians in deciding the best empirical treatment for a patient. Identifying the most probable site of infection and considering the resistance rate to an antibiotic regarding the culture sample collected could help in deciding on a certain antibiotic treatment besides the suspected bacteria. In clinical settings, in a septic patient, the clinician should not expect an antibiogram result, but should initiate an empirical treatment as soon as possible [33,34].

The analysis of bacterial resistances is another important aspect of our study. Our data show that *Escherichia coli* present the highest resistance rates to ampicillin and trimethoprim-sulfamethoxazole. For instance, in a study by Draganescu from 2016, the highest resistance rates of *Escherichia coli* were to amoxicillin–clavulanic acid and ciprofloxacin [35]. On the other hand, recent data published by Kun Li show a much higher resistance rate for *Escherichia coli*, with up to 87.7% for ampicillin, more than 50% for cephalosporines and 30–35% for quinolones and aminoglycosides [25]. Regarding the global burden of bacterial antimicrobial resistance in 2019, it was estimated that the *Escherichia coli* resistance rate to third-generation cephalosporin and fluoroquinolones was between 20 and 30%, much higher than our findings [1]. 

*Staphylococcus aureus* presented the highest resistance rates to clindamycin and oxacillin. In a recent study by Talapan, the *Staphylococcus aureus* resistance rate to oxacillin was higher (39%) but closer regarding clindamycin (36%) [36]. On the other hand, in a study by Kaur, the resistance rate of methicillin-resistant *Staphylococcus aureus* was up to 100% for oxacillin, clindamycin, quinolones and aminoglycosides [37].

*Klebsiella* spp. presented the highest resistance rates to piperacillin-tazobactam (25.2%), ampicillin (20.4%) and cefuroxime (14.8%). The resistance analysis of *Klebsiella* from another Romanian hospital showed higher resistance rates (36% to piperacillin-tazobactam and over 50% to cephalosporins) [14]. For instance, the worldwide analysis of *Klebsiella pneumoniae* estimates that the resistance rate is 30–40% for pemens and 60–70% for third-generation cephalosporins [1]. The 2019 global burden analysis estimated that in Romania, more than 80% of *Acinetobacter baumanii* strains are resistant to penems [1], which is close to our results.

One should keep in mind that differences regarding resistance could be influenced by different sensitivities in the techniques. Regarding Romania, the results are comparable given the fact that the same identification methods are used for bacteria or for antimicrobial resistance quantification.

As already mentioned, the antibiotic resistance patterns differ from region to region. There are several factors that influence resistance, and the most common ones are represented by abnormal or abusive antibiotic use not only in patients, but even in agriculture, the food industry and through environmental contamination [38,39,40,41,42].

Our study has several limitations. First, this is a single-center study; thus, our results mainly represent our study region. The fact that this is a cross-sectional study without patient follow-up or a dynamic strain resistance evaluation is another drawback. Another important limitation is the lack of uniform antibiotic testing for the same strains. The strong points are the evaluation of an entire year of positive cultures and the relatively high number of positive cultures.

## 5. Conclusions

In the clinical setting, the highest frequencies of positive cultures were urocultures, wound secretion cultures and blood cultures. Our results are in concordance with previously published data, with *Escherichia coli*, *Staphylococcus aureus* and *Klebsiella* spp. being the most common ones. Overall, our results demonstrate lower resistance rates compared with those in the literature, especially for *Escherichia coli*, *Staphylococcus aureus* and *Klebsiella* spp. The clinical impact of our study is mainly local, and our results could guide clinicians in deciding on an empirical treatment when they suspect a certain infection site and/or specific bacteria. The results should be cautiously interpreted as they represent the west part of Romania. On the other hand, our results are a reflection of the current antimicrobial resistance patterns depending on the infection site and age. Larger multi-center prospective studies are needed in order to reduce the global burden of misguided antibiotic use. 

## Figures and Tables

**Table 1:** Recorded antibiotics.

Antibiotic Class	Antibiotic
Aminoglycozides	Gentamicin
	Amikacin
Carbapenems	Meropenem
	Imipenem
Fluoroqinolones	Ciprofloxacin
	Levofloxacin
Cephalosporins	Cefepim
	Ceftriaxone
	Ceftazidime
	Cefuroxime
Lincosamides	Clindamycin
Glicopeptides	Vancomycin
Penicilines	Amoxicillin–clavulanic acid
	Ampicillin
	Oxacillin
	Piperacillin-tazobactam
Monobactames	Aztreonam
Polimixines	Colistin
Sulfonamides	Trimethoprim-sulfamethoxazole
Oxazolidinones	Linezolid

**Table 2:** The age distributions of the patients with positive cultures.

Age Group	Number of Patients	Percentage (%)
0–9 years	249	6.97
10–19 years	85	2.32
20–29 years	227	6.36
30–39 years	302	8.25
40–49 years	313	8.55
50–59 years	492	13.44
60–69 years	862	23.56
70–79 years	728	19.9
80–89 years	343	9.37
90–100 years	57	1.55

**Table 3:** Bacteria distribution stratified by culture type.

	Number of Cultures	Number of Identified Strains	PA	EN	SA	BC	KL	ST	AB	SM	PROV	PROT	CO	CI	EC
Uroculture	1343	1098	57	19	37	0	209	83	10	14	6	72	1	5	585
Wound secretion culture	738	885	121	10	313	17	93	16	27	21	6	91	1	10	84
Blood culture	360	381	29	25	189	2	28	12	14	1	1	7	4	1	51
Bronchial aspirate culture	229	235	28	16	37	0	43	11	65	1	2	9	3	2	18
Cervical culture	214	222	4	2	22	0	23	56	1	1	0	7	0	0	106
Sputum culture	125	122	26	7	27	0	32	11	2	0	0	3	0	0	14
Abscess culture	113	122	2	1	18	0	29	10	0	2	1	8	0	1	50
Vaginal culture	92	110	9	9	26	0	7	11	0	0	0	6	0	0	42
Peritoneal liquid culture	82	80	6	5	8	0	13	10	1	2	0	3	0	2	42
Catheter tip culture	42	44	4	5	21	1	4	1	3	0	0	1	0	0	4
Total	3338	3299	286	99	698	20	481	221	123	42	16	207	9	21	996
Percentages			8.56	2.96	20.9	0.59	14.4	6.62	3.68	1.25	0.47	6.2	0.26	0.62	29.83

Legend: PA = *Pseudomonas aeruginosa*, EN = *Enterobacter*, SA = *Staphylococcus aureus*, BC = *Bacillus cereus*, KL = *Klebsiella* spp., ST= *Streptococcus*, AB= *Acinetobacter baumanii*, SM= *Serratia marcesnens*, PROV= *Providencia*, PROT= *Proteus*, CO = *Corynebacterium*, CI = *Citrobacter*, EC = *Escherichia coli*.

## Data Availability

The original contributions presented in this study are included in the article/Supplementary Material, and further inquiries can be directed to the corresponding author at the e-mail address marc.luciana@umft.ro. The raw data supporting the conclusions of this article will be made available by the authors upon request. All of the data are presented in the current form of the manuscript.

## Supplementary Materials

The following supporting information can be downloaded at: https://www.mdpi.com/article/10.3390/microorganisms12081517/s1, Supplemental Table S1—the distribution of resistances regarding the evaluated antibiotics stratified by cultures; Supplemental Table S2—the distribution of sensibilities regarding the evaluated antibiotics stratified by cultures; Supplemental Table S3—the distribution of resistances regarding the evaluated antibiotics stratified by bacteria; Supplemental Table S4—the distribution of sensibilities regarding the evaluated antibiotics stratified by bacteria.
