# Supplementary material for: Bacterial Resistances and Sensibilities in a Tertiary Care Hospital in Romania—A Retrospective Analysis
Source: Microorganisms. 2024 Jul 24;12(8):1517. doi: 10.3390/microorganisms12081517 (PMC11356133; doi:10.3390/microorganisms12081517)
Supplement: Supplementary file 1 [file microorganisms-12-01517-s001.zip › microorganisms-3097411-supplementary.pdf]

Supplemental table 1 – the distribution of resistances regarding the evaluated antibiotics stratified by cultures.

Culture	Nr. of strains	GEN	AMI	MER	IMI	CIP	LEV	CEF	CFX	CFT	CFR	CLI	VAN	AMO	AMP	OXA	PIP	AZT	COL	TRI	LIN
Uroculture	1098	12.1	**2**	**1.6**	**1.3**	**19**	**16.8**	**6**	**8.1**	8.3	**10.6**	**1.9**	0	2.8	**18**	**0.8**	**13**	**0.07**	**0.2**	**12.7**	0
Wound secretion culture	885	13.4	3.1	2.7	4.7	16.4	12.6	**5**	6.8	**6.9**	7.5	**14.6**	0	**3.9**	13.1	**9.3**	12.7	**0.1**	0.4	10.8	0
Blood cultures	381	**19.2**	1.9	2.8	3.6	**21.1**	13.3	8.1	**1.9**	8.6	**1.9**	8.1	0	2.2	**5.6**	**9.2**	**3.3**	**5.8**	0.3	**5.8**	0
Bronchial aspirate culture	235	**32.8**	**14.4**	**27.1**	**28.8**	**36.7**	13.5	**29.7**	5.2	**34.5**	**2.6**	4.8	0	1.7	**8.3**	2.2	**14.8**	**11.8**	**5.2**	7	0
Cervical culture	222	**4.2**	**0.**	**0**	**O**	**2.8**	**5.1**	**2.8**	4.2	**3.7**	4.2	7.9	0	**0**	9.8	2.8	8.9	**0**	0	7	0
Sputum culture	122	10.4	3.2	1.6	2.4	**9.6**	**6.4**	4	5.6	6.4	8	10.4	0	0.8	8.8	6.4	10.4	0	0	4.8	0
Abscess culture	122	8.8	2.7	1.8	1.8	13.3	8	6.2	6.2	6.2	5.3	8.8	0	0	8.8	4.4	8.8	0.9	0	14.2	0
Vaginal culture	110	**3.3**	0	0	0	**2.2**	**2.2**	**1.1**	**1.1**	**1.1**	**1.1**	5.4	0	1.1	6.5	3.3	**2.2**	0	0	1.1	0
Peritoneal liquid culture	80	9.9	0	1.4	2.8	16.9	9.9	**15.5**	1.4	15.5	1.4	**1.4**	0	0	**4.2**	0	4.2	**14.1**	0	4.2	0
Catheter tip culture	44	**35.7**	2.4	2.4	2.4	**42.9**	**31**	9.5	11.9	9.5	4.8	**16.7**	0	**7.1**	14.3	**19**	14.3	0	0	**26.2**	0
Overall	3299	13.2	2.8	3.4	4	16.9	12.3	7.3	5.9	9.3	6.9	8.1	0	2.4	12.4	4.8	10.8	2.2	0.6	9.9	0

Legend: The bolded values presented statistical significant differences when compared with means ($p<0.05$). Values starting with the 3rd column represent percentages. The underlined ones presented higher percentages of resistance and non-underlined ones lower. Nr.=number, GEN=gentamycin, AMI=amikacin, MER=meropenem, IMI=imipenem, CIP=ciprofloxacin, LEV=levofloxacin, CEF=cefepim, CFX=ceftriaxone, CFT=ceftazidime, CFR=cefuroxime, CLI=clindamycin, VAN=vancomycin, AMO=amoxicillin-clavulanic acid, AMP=ampicillin, OXA=oxacillin, PIP=piperacilline-tazobactam, AZT=aztreonam, COL=colistin, TRI=trimetoprim-sulfametoxazol, LIN=linezolid.

Supplemental table 2 – the distribution of sensibilities regarding the evaluated antibiotics stratified by cultures.

Culture	Nr. of strains	GEN	AMI	MER	IMI	CIP	LEV	CEF	CFX	CFT	CFR	CLI	VAN	AMO	AMP	OXA	PIP	AZT	COL	TRI	LIN
Uroculture	1098	63.7	**44.9**	39.2	**43.4**	**37.9**	**35.9**	29.4	**41.8**	**36.5**	**40**	**3.4**	12.4	**18.4**	**52.9**	**1.6**	**35.7**	**0**	**14.7**	35	**22.9**
Wound secretion culture	885	**71.4**	**45.7**	39.4	**32.1**	**59.1**	35.2	32	**28.3**	31.6	**20.2**	**21.8**	**15.4**	**19.4**	34.7	**22.8**	**38.2**	**0.7**	14.9	**39.2**	**58**
Blood cultures	381	60.6	**11.1**	**25.3**	**24.2**	48.3	23.3	**18.9**	**7.2**	**20.3**	**5.8**	**15.3**	**31.9**	**1.9**	**8.3**	10	**8.1**	**8.1**	19.7	**20**	**56.9**
Bronchial aspirate culture	235	**40.6**	**21.8**	**29.7**	**26.6**	**31**	**11.8**	**22.3**	**6.1**	**24.9**	**5.2**	**5.7**	9.6	1.7	**3.5**	**3.5**	**16.2**	**9.6**	**41**	**11.8**	**22.3**
Cervical culture	222	65	**60.7**	**50.9**	**48.6**	49.1	**51.9**	**44.4**	**72**	**43.5**	**51.9**	**18.7**	**0.5**	**23.8**	**61.7**	**4.7**	**53.7**	**0**	15.4	38.8	34.1
Sputum culture	122	**48**	46.4	**28.8**	30.4	41.6	**22.4**	25.6	**22.4**	28.8	20.8	9.6	0	**4.8**	**23.2**	9.6	**41.6**	0.8	8	28	**22.4**
Abscess culture	122	71.7	**62.8**	**59.3**	**53.1**	**60.2**	**48.7**	**45.1**	**53.1**	**46.9**	**47.8**	13.3	8	18.6	**51.3**	8	**46.9**	3.5	18.6	33.6	34.5
Vaginal culture	110	70.7	41.3	38	46.7	**57.6**	35.9	29.3	**52.2**	39.1	**48.9**	15.2	**1.1**	10.9	**56.5**	7.6	35.9	0	**8.7**	**53.3**	30.4
Peritoneal liquid culture	80	74.6	38	**67.6**	**64.8**	**60.6**	31	**47.9**	28.2	**49.3**	21.1	**4.2**	15.5	14.1	33.8	**0**	31	**21.1**	19.7	**14.1**	31
Catheter tip culture	44	**35.7**	**21.4**	**21.4**	**21.4**	**28.6**	**14.3**	**14.3**	**7.1**	**16.7**	**9.5**	9.5	**28.6**	7.1	**9.5**	2.4	**11.9**	0	19	23.8	**61.9**
Overall	3299	63.9	39.9	38	37.3	47.4	32.6	29.7	32.7	32.7	27.9	12	12.8	14.7	37.7	9.8	32.5	2.8	16.6	33.8	38.2

Legend: The bolded values presented statistical significant differences when compared with means ($p<0.05$). Values starting with the 3rd column represent percentages. The underlined ones presented higher percentages of resistance and non-underlined ones lower. Nr.=number, GEN=gentamycin, AMI=amikacin, MER=meropenem, IMI=imipenem, CIP=ciprofloxacin, LEV=levofloxacin, CEF=cefepim, CFX=ceftriaxone, CFT=ceftazidime, CFR=cefuroxime, CLI=clindamycin, VAN=vancomycin, AMO=amoxicillin-clavulanic acid, AMP=ampicillin, OXA=oxacillin, PIP=piperacilline-tazobactam, AZT=aztreonam, COL=colistin, TRI=trimetoprim-sulfametoxazol, LIN=linezolid.

Supplemental table 3 – the distribution of resistances stratified by bacteria

Culture	Nr. of strains	GEN	AMI	MER	IMI	CIP	LEV	CEF	CFX	CFT	CFR	CLI	VAN	AMO	AMP	OXA	PIP	AZT	COL	TRI	LIN
Acinetobacter baumanii	123	**53.9**	**23.4**	**52.3**	**48.4**	**56.2**	**22.7**	**46.9**	9.4	**53.9**	10.9	**2.3**	0	1.6	10.9	**0.8**	**35.2**	0.8	1.6	12.5	0
Bacillus cereus	20	0	0	0	0	5.3	5.3	0	**36.8**	0	0	5.3	0	**57.9**	**57.9**	5.3	0	0	0	**26.3**	0
Citrobacter	21	11.1	0	3.7	**14.8**	7.4	7.4	14.8	11.1	**25.9**	11.1	0	0	0	11.1	0	18.5	**11.1**	0	7.4	0
Corynebacterium	9	0	0	0	0	0	**44.4**	0	**55.6**	0	0	**55.6**	0	**22.2**	**55.6**	0	0	0	0	0	0
Enterobacter spp.	99	12	5	3	3	15	9	**15**	10	**20**	4	6	0	1	10	5	14	**12**	1	11	0
Escherichia Coli	996	**6.7**	**1**	**0.4**	**0.8**	**14.1**	**9**	8.2	**8.4**	10.2	**12**	**1.45**	0	1.7	**19.8**	**0.8**	**14.5**	**3**	**0.2**	**16.4**	0
Klebsiella spp	481	12	3.6	4.8	**6.2**	16.8	12	**15.2**	**13.4**	**17.8**	**14.8**	**1.8**	0	**1**	**20.4**	**0.8**	**25.2**	**6.4**	**1.2**	**13.2**	0
Proteus	207	17.4	3.3	1.4	**7.5**	14.6	10.3	5.2	**8.9**	11.7	**12.2**	**4.2**	0	3.8	**18.3**	2.3	13.6	1.4	0.5	**16.9**	0
Providencia	16	**52.9**	**47.1**	**35.3**	**29.4**	**52.9**	**41.2**	**29.4**	**29.4**	**52.9**	**23.5**	0	0	0	**35.3**	0	**47.1**	5.9	**5.9**	**41.2**	0
Pseudomonas aeruginosa	286	14.9	**8.6**	**8.3**	**12.9**	17.2	14.6	**10.3**	**3**	**14.6**	**3.3**	**4**	0	**0.7**	**7.6**	3	**16.9**	3	**1.7**	**6.6**	0
Serratia marcesnens	42	19	4.8	7.1	7.1	**28.6**	**23.8**	14.3	9.5	14.3	9.5	2.4	0	0	11.9	0	19	4.8	**4.8**	16.7	0
Staphylococcus aureus	698	13.5	**0.6**	**0.6**	**0.9**	**14.2**	**7.7**	**1.2**	**1.1**	**1.5**	**1.6**	**27.4**	0	**1.2**	**4.6**	**19.7**	**3**	**0.3**	**0.1**	8.6	0
Streptococcus spp	221	**9.9**	**0.5**	**0**	**0.3**	**4.9**	10.7	**0.8**	**1.6**	**1.1**	**1.4**	**24.4**	0	1.4	**9**	**7.9**	**2.2**	**0.5**	0	**5.2**	0
Overall	3299	13.2	2.8	3.4	4	16.9	12.3	7.3	5.9	9.3	6.9	8.1	0	2.4	12.4	4.8	10.8	2.2	0.6	9.9	0

Legend: The bolded values presented statistical significant differences when compared with means ($p<0.05$). Values starting with the 3rd column represent percentages. The underlined ones presented higher percentages of resistance and non-underlined ones lower. Nr.=number, GEN=gentamycin, AMI=amikacin, MER=meropenem, IMI=imipenem, CIP=ciprofloxacin, LEV=levofloxacin, CEF=cefepim, CFX=ceftriaxone, CFT=ceftazidime, CFR=cefuroxime, CLI=clindamycin, VAN=vancomycin, AMO=amoxicillin-clavulanic acid, AMP=ampicillin, OXA=oxacillin, PIP=piperacilline-tazobactam, AZT=aztreonam, COL=colistin, TRI=trimetoprim-sulfametoxazol, LIN=linezolid.

Supplemental table 4 – the distribution of sensibilities stratified by bacteria

Culture	Nr. of strains	GEN	AMI	MER	IMI	CIP	LEV	CEF	CFX	CFT	CFR	CLI	VAN	AMO	AMP	OXA	PIP	AZT	COL	TRI	LIN
Acinetobacter baumanii	123	**28.9**	**21.1**	**21.9**	**19.5**	**23.4**	**14.8**	**18.8**	**5.5**	32.8	**5.5**	**2.3**	**4.7**	**7.8**	**10.9**	**1.6**	**16.4**	0.6	**75**	**16.4**	**10.9**
Bacillus cereus	20	0	**10.5**	**5.3**	**5.3**	68.4	21.1	5.3	**10.5**	5.3	**0**	**84.2**	**78.9**	26.3	31.6	5.3	**10.5**	0	0	26.3	**100**
Citrobacter	21	84.2	**77.8**	**88.9**	**85.2**	**92.6**	**51.9**	**74.1**	37	**66.7**	**51.9**	11.1	**0**	7.4	37	11.1	**59.3**	**22.2**	**33.3**	48.1	**14.8**
Corynebacterium	9	44.4	**0**	11.1	11.1	**11.1**	**0**	11.1	11.1	11.1	0	11.1	0	**55.6**	11.1	11.1	**0**	11.1	11.1	**0**	**77.8**
Enterobacter spp.	99	71	5	**54**	**55**	**61**	28	**44**	27	**48**	22	6	**5**	**3**	**12**	**4**	31	**17**	**26**	37	**17**
Escherichia Coli	996	**78.9**	41	**65.8**	**69.6**	**58.5**	**43.6**	**50.9**	**8.4**	**56.7**	**64**	**1.5**	**1.7**	**4.8**	**57.2**	**1.4**	**55.6**	**4.1**	22.1	**45.7**	**6.4**
Klebsiella spp	481	**69.3**	**68.9**	**64.1**	**61.9**	**57.1**	**40.1**	**44.3**	**61.2**	**50.9**	**40.5**	**2**	0.6	3.4	39.7	**2**	**52.1**	**6.4**	**32.3**	**43.7**	**6.6**
Proteus	207	62	**77.9**	**70**	59.6	56.3	**44.6**	**57.7**	**55.9**	**51.6**	**54**	**1.9**	**4.7**	**9.4**	**63.8**	**4.7**	**70.4**	1.9	9.4	38	**16**
Providencia	16	**23.5**	29.4	58.8	23.5	**17.6**	**5.9**	41.2	17.6	29.4	**0**	0	5.9	5.9	**5.9**	0	35.3	5.9	17.6	11.8	**5.9**
Pseudomonas aeruginosa	286	67.2	**63.9**	**58.6**	**49**	**59.9**	**42.7**	**54**	**14.2**	**58.3**	**9.9**	**5**	**4.6**	**5**	**13.6**	**5**	**58.3**	4	**37.7**	**15.6**	**16.6**
Serratia marcesnens	42	73.8	**81**	**83.3**	**78.6**	**71.4**	**54.8**	**69**	**59.5**	**71.4**	21.4	7.1	**2.4**	**2.4**	**16.7**	7.1	**64.3**	4.8	19	**52.4**	**14.3**
Staphylococcus aureus	698	**68.2**	**11.1**	**9.9**	**9.1**	**57.9**	**19.1**	**8.2**	**9.7**	**8.9**	**6.7**	**35.6**	**18.5**	**5.5**	**10.3**	**39.6**	**9.4**	**0.8**	**3.4**	**49.2**	**89.5**
Streptococcus spp	221	**47.1**	**4.4**	**5.5**	**6.8**	30.7	32.9	**4.4**	**49**	**5.8**	**5.5**	**43.3**	**6**	**46.8**	**46.8**	**16.2**	**4.4**	**0.3**	**2.5**	27.7	**83.6**
Overall	3299	63.9	39.9	38	37.3	47.4	32.6	29.7	32.7	32.7	27.9	12	12.8	14.7	37.7	9.8	32.5	2.8	16.6	33.8	38.2

Legend: The bolded values presented statistical significant differences when compared with means (p<0.05). Values starting with the 3rd column represent percentages. The underlined ones presented higher percentages of resistance and non-underlined ones lower. Nr.=number, GEN=gentamycin, AMI=amikacin, MER=meropenem, IMI=imipenem, CIP=ciprofloxacin, LEV=levofloxacin, CEF=cefepim, CFX=ceftriaxone, CFT=ceftazidime, CFR=cefuroxime, CLI=clindamycin, VAN=vancomycin, AMO=amoxicillin-clavulanic acid, AMP=ampicillin, OXA=oxacillin, PIP=piperacilline-tazobactam, AZT=aztreonam, COL=colistin, TRI=trimetoprim-sulfametoxazol, LIN=linezolid.
